# The 2022 Vaccines Against *Shigella* and Enterotoxigenic *Escherichia coli* (VASE) Conference: Summary of breakout workshops

**DOI:** 10.1016/j.vaccine.2023.11.045

**Published:** 2024-03-07

**Authors:** Shahida Baqar, Aurelio Bonavia, A. Louis Bourgeois, Joseph J. Campo, Allison Clifford, Kurt Hanevik, Mateusz Hasso-Agopsowicz, William Hausdorff, Robert Kaminski, Calman A. MacLennan, Nicholas Mantis, Laura B. Martin, Richard Omore, Marcela Pasetti, Patricia Pavlinac, Armelle Phalipon, Frédéric Poly, Chad Porter, Maheshi N. Ramasamy, Elizabeth T. Rogawski McQuade, Marcelo B. Sztein, Richard Walker

**Affiliations:** a US National Institutes of Health, United States; b Bill & Melinda Gates Medical Research Institute, United States; c PATH, United States; d Antigen Discovery, Inc, United States; e University of Bergen, Norway; f Norwegian National Advisory Unit for Tropical Infectious Diseases, Medical Department, Haukeland University Hospital, Norway; g World Health Organization, Switzerland; h Latham BioPharm Group, United States; i Enteric and Diarrheal Diseases, Bill & Melinda Gates Foundation, United Kingdom; j The Jenner Institute, United Kingdom; k Wadsworth Center, New York State Department of Health, United States; l US Pharmacopeial Convention, United States; m Kenya Medical Research Institute Center for Global Health Research, Kenya; n University of Maryland, United States; o University of Washington, United States; p Institut Pasteur, France; q Naval Medical Research Command, United States; r Oxford Vaccine Group, University of Oxford, United Kingdom; s Emory University, United States; t Faculty of Medicine, Université Libre de Bruxelles, Belgium

**Keywords:** Enteric diseases, Vaccine development, *Shigella*, Enterotoxigenic *Escherichia coli*, *Campylobacter*, Invasive non-typhoidal *Salmonella*

## Abstract

The global public health nonprofit organization PATH hosted the third Vaccines Against *Shigella* and Enterotoxigenic *Escherichia coli* (VASE) Conference in Washington, DC, from November 29 to December 1, 2022. This international gathering focused on cutting-edge research related to the development of vaccines against neglected diarrheal pathogens including *Shigella*, enterotoxigenic *Escherichia coli* (ETEC), *Campylobacter*, and non-typhoidal *Salmonella*. In addition to the conference’s plenary content, the agenda featured ten breakout workshops on topics of importance to the enteric vaccine field. This unique aspect of VASE Conferences allows focused groups of attendees to engage in in-depth discussions on subjects of interest to the enteric vaccine development community. In 2022, the workshops covered a range of topics. Two focused on the public health value of enteric vaccines, with one examining how to translate evidence into policy and the other on the value proposition of potential combination vaccines against bacterial enteric pathogens. Two more workshops explored new tools for the development and evaluation of vaccines, with the first on integrating antigen/antibody technologies for mucosal vaccine and immunoprophylactic development, and the second on adjuvants specifically for *Shigella* vaccines for children in low- and middle-income countries. Another pair of workshops covered the status of vaccines against two emerging enteric pathogens, *Campylobacter* and invasive non-typhoidal *Salmonella*. The remaining four workshops examined the assessment of vaccine impact on acute and long-term morbidity. These included discussions on the nature and severity of intestinal inflammation; cellular immunity and immunological memory in ETEC and *Shigella* infections; clinical and microbiologic endpoints for *Shigella* vaccine efficacy studies in children; and intricacies of protective immunity to enteric pathogens. This article provides a brief summary of the presentations and discussions at each workshop in order to share these sessions with the broader enteric vaccine field.

## Highlights

• Economic evaluations of enteric vaccine impact will help guide health investments.
• Advancing adjuvant-vaccine combinations to human clinical trials is critical.
• Emerging enteric pathogens like iNTS and *Campylobacter* need further advocacy.
• New tools for characterizing inflammation are needed to improve health outcomes.
• New technology to characterize adaptive immunity makes more protective vaccines.

## 1 Introduction

To help accelerate the development of vaccines against *Shigella*, enterotoxigenic *Escherichia coli* (ETEC), and other neglected enteric pathogens, the global nonprofit organization PATH convenes the Vaccines Against *Shigella* and ETEC (VASE) Conference. The third VASE Conference was held in Washington, DC, on November 29 to December 1, 2022. In addition to plenary content (summarized elsewhere in this issue of *Vaccine*), the agenda featured ten workshops on topics of importance to the enteric vaccine field. Breakout workshops are a distinctive aspect of every VASE Conference and allow focused groups of attendees to engage in deeper discussions on particular topics.

PATH holds an open call for individuals to propose workshop concepts on subjects of interest to the enteric vaccine development field. Each workshop is unique as the organizers must independently develop an agenda and plan presentations and discussion time. In 2022, the workshops covered four broad topic areas: the public heath value of vaccines; new tools for the development and evaluation of vaccines; emerging enteric pathogens; and the assessment of vaccine impact on acute and long-term morbidity. Table 1 provides a complete list of the workshops by topic area conducted during the conference and brief key takeaways from each one. The article that follows further expands on these key points and offers a detailed summary of the presentations and discussions from each workshop in order to share these sessions with the broader enteric vaccine field.

**Table 1:** List of workshops conducted at the 2022 VASE Conference by topic area and related key takeways.

**Topic area**	**Workshop name**	**Key takeaways**
The public health value of vaccines	The full value of vaccines against enteric pathogens: How to translate evidence into policy	• Introduction of existing enteric vaccines has been slow, but clearer assessment and appreciation of the full public heath value of vaccines by stakeholders could translate into increased use. • Challenges remain about how best to influence country decisions about introduction and use of vaccines. Audience-tailored messages using regional data and clear assumptions are critical to increasing awareness of the benefits of vaccination on health, as well as physical and cognitive development.
	The value proposition of combination vaccines targeted for use against bacterial enteric pathogens	• It is likely that significant uptake of new vaccines in low- and middle-income countries, as well as among at-risk travelers (dual markets), will require protective antigens against multiple pathogens to be grouped in combination formulations. • The lack of clear policy guidance and financing mechanisms for combination vaccines, along with complex clinical testing and regulatory pathways, represent the greatest challenges to combination vaccine development.

New tools for the development and evaluation of vaccines	Integrating antigen/antibody technologies for mucosal vaccine and immunoprophylactic development	• There is a need for broader immune profiling to account for variations across cohorts when defining correlates of protection and anticipated impact of vaccines on disease incidence; a single immune parameter may be insufficient to define protection. • Protective responses that are common across populations may be better predictors of protection than antibody levels alone.
	The need for adjuvants for *Shigella* vaccines to ensure protective efficacy among children in LMICs	• Adjuvants should be prioritized based on ability to formulate, enhancement of vaccine immunogenicity, and cost. • Vaccines formulated with novel adjuvants should be tested in animal models and early-stage clinical trials without waiting for new clinical data on the adjuvants alone, which could result in lost opportunities for significant product improvement and innovation.

Emerging enteric pathogens	Invasive non-typhoidal salmonellosis: Developing a vaccine against a neglected disease	• Development of an effective and affordable vaccine against invasive non-typhoidal salmonellosis (iNTS) is an essential control measure against this neglected disease. • An effective bivalent *Salmonella* Enteritidis and *Salmonella* Typhimurium vaccine would clearly target iNTS disease in children in low- and middle-income countries.
	Prevention strategies to defeat *Campylobacter*	• Increased advocacy and education on the burden of *Campylobacter* to country-level health decision-makers would be required to support the future introduction of either passive and or active prophylactic measures. • The development of a pan-diarrhea vaccine that covers the most common causes of pediatric diarrheal disease including *Campylobacter* would likely be more accepted than a standalone vaccine.

The assessment of vaccine impact on acute and long-term morbidity	Characterizing the nature and severity of intestinal inflammation to guide enteric vaccine and prophylactic product development and evaluation	• Continued evaluation of new tools for characterizing inflammation will improve understanding of the complex interplay between intestinal and systemic inflammation that leads to poor health outcomes in young children. • An index of multiple biomarkers may be needed to adequately characterize the risk of negative health outcomes and suboptimal immunization responses associated with aberrant gut and systemic inflammation.
	Cellular immunity and immunological memory in ETEC and *Shigella* infections and vaccination in humans	• Our understanding of the kinetics and functioning of immune cell subsets in protection from ETEC and *Shigella* remains inadequate. • The role of T-cell and B-cell subsets in innate and adaptive immunity needs to be better defined and their association with protection assessed to guide the development of vaccines that can modulate the immune response toward durable protection.
	Clinical and microbiologic endpoints for *Shigella* vaccine efficacy studies in children in LMICs	• Various clinical and microbiological endpoints could be considered for *Shigella* vaccine efficacy studies, all of which have sample size implications. • In addition to the reduction of disease, the effects of a *Shigella* vaccine candidate on intestinal and systemic inflammation, linear growth faltering, mortality, and antimicrobial use and resistance also need to be understood.
	Intricacies of protective immunity to enteric pathogens	• There is a continued need to advance the availability and use of reagents and standards to allow the global community to better interrogate protective immune mechanisms to enteropathogens. • The identification of additional immunogenic targets for vaccine development and improved understanding of the complexities and elusiveness of immunity to enteric pathogens are critical.

## 2 The public health value of vaccines

### 2.1 The full value of vaccines against enteric pathogens: How to translate evidence into policy

#### Organizer: Mateusz Hasso-Agopsowicz (World Health Organization)

Vaccines against enteric diseases such as rotavirus, typhoid, or cholera are already licensed, however, their introduction has been slow. In this workshop, several speakers and a panel of experts deliberated with the audience on why this is the case and how assessing the full value of vaccines could translate into increased use.

The global coverage of rotavirus vaccines is at 49 % and typhoid conjugate vaccine has only recently been introduced in a handful of countries [1], [2]. Vaccines in development against *Shigella* spp. and ETEC are expected to be used in low- and middle-income countries (LMICs) where disease burden is highest and return on investment limited. The development of enteric vaccines and subsequent policy decision and country uptake are at risk due to a decrease in enteric mortality, limited commercial incentive to develop vaccines for use in LMICs, and the need to expand busy immunization schedules with multiple injections. As such, one must recognize that vaccines not only avert infections and deaths but can also reduce other disease-related outcomes such as long-term disability, like stunting and enteric environmental dysfunction, antimicrobial resistance (AMR), disease outbreaks, and economic burden like cost of treatment or family and societal disruption. Such an assessment to capture the full value of vaccines is critical to prioritize investment for vaccine development, collect data for policy decisions, and convince countries to introduce and use enteric vaccines.

Mortality in children under five is often a priority component of the full value of vaccines, however, varied estimates have been reported for ETEC and *Shigella*. As such, the World Health Organization (WHO) and enteric disease experts have assessed the reported estimates and conducted analyses to fill data gaps, such as a systematic review and analyses of association of pathogen detection with diarrhea, or case fatality rates reported by age and region [3], [4], [5]. Additionally, the group has published recommendations for studies to be included when estimating the burden of enteric diseases [6].

To optimize the impact of vaccines on AMR, WHO published a framework to accelerate the use of licensed vaccines to reduce AMR and antibiotic use, develop new vaccines with potential impact on AMR, and collect and share data [7]. The analysis of a pipeline of vaccine candidates in clinical trials found that there are six vaccine candidates each for *Shigella* and ETEC [8]. Modeling studies suggest that a wide use of rotavirus vaccines in LMICs could avert 32 million antibiotic-treated episodes annually [9]. Recently, typhoid conjugate vaccine introduced to deal with the outbreak of extensively drug-resistant (XDR) typhoid in Pakistan was found to be more than 94 % efficacious in preventing all typhoid and XDR typhoid [10]. Additional analyses show that vaccines can reduce infections and deaths caused by AMR enteric pathogens [11].

Economic evaluations of vaccine impact aid in decision-making for appropriate health investments. Standard measures include cost per disability-adjusted life year averted, often compared to country-specific GDP per capita. For enteric vaccines, one must conduct comprehensive analyses that consider a broader set of benefits of vaccination, including longer-term morbidity such as stunting and non-communicable diseases, along with the potential economic benefits that could arise from their reduction. One study showed that a vaccine against *Shigella*, which could prevent stunting, would be both cost-effective and cost-saving in the longer-term [12], [13].

Challenges remain about translating evidence into policy and how best to influence country decisions about introduction and use of vaccines. Audience-tailored messages using regional data and clear assumptions are critical. These could be delivered as country or regional workshops to build awareness of pathogen burden and vaccine impact. Presenting a full value of vaccines is critical to policymakers and country stakeholders, as it is often difficult to predict what drives a decision on vaccine policy and introduction. One of the drivers for rotavirus introduction outside of LMICs was the high rate of hospitalization during winter, for meningitis it was the need to control outbreaks, and a vaccine against Japanese encephalitis was introduced to reduce the long-term morbidity associated with the disease.

### 2.2 The value proposition of combination vaccines targeted for use against bacterial enteric pathogens

#### Organizer: William Hausdorff (PATH)

The public health importance of preventing disease caused by *Shigella,* the most prominent *Salmonella* pathogens (typhoid, paratyphoid, and invasive non-typhoidal *Salmonella*), ETEC*,* and *Campylobacter* is well established, and in many cases promising vaccine candidates are either licensed (typhoid) or in advanced clinical development (ETEC). However, in light of the increasingly crowded immunization schedule, and the limited tolerance of caregivers and even healthcare workers for multiple injections given to children at each visit, it seems likely that significant vaccine uptake in LMICs will only occur if protective antigens against multiple pathogens are grouped in combination formulations. The challenges and opportunities of designing, developing, testing, and licensing these combinations was the subject of this workshop.

Mateusz Hasso-Agopsowicz (WHO) presented the practical and epidemiological rationale for combination vaccine development, noting that as enteric bacterial vaccine impact on mortality will be lower than existing vaccines, and that novel delivery devices, articulating the full value of vaccines, and combination vaccines could each help to introduce and deliver vaccines in LMICs. He cited the precedent of Hib and rubella vaccines, whose introduction was driven by large investments by Gavi, the Vaccine Alliance, once they were included within larger combination formulations.

Bill Hausdorff highlighted considerations regarding combination vaccines containing *Shigella* which included a multitude of epidemiological, clinical development, regulatory, manufacturing, policy, and financial aspects [14]. Workshop participants agreed that the lack of clear global or regional policy guidance and financing mechanisms for combination vaccines, along with potentially highly complex clinical testing and regulatory pathways, may represent the greatest challenges to *Shigella* combination vaccine development.

Presenting on behalf of Mark Riddle (University of Nevada, Reno School of Medicine), Lou Bourgeois (PATH) described the results of surveys of travel medicine providers and interviews with military personnel involved in immunization policy. They concluded that a combination vaccine appears attractive to both markets given the polymicrobial nature of travelers’ diarrhea, but a consensus is yet to be reached on its optimal composition. Given the overlap in etiology and epidemiology of infectious diarrhea and other enteric diseases in high-income vs. LMICs, it was concluded that combination vaccines could have a significant “dual market” potential that would likely help ensure better uptake and impact [45].

Based on a mixed-methods interview-based survey of 89 LMIC national stakeholders and healthcare providers in Nepal, Vietnam, Kenya, Malawi, and Burkina Faso, Jessica Fleming (PATH) discussed the potential interest in and demand for *Shigella*-containing combination vaccines in LMICs. She noted that although nearly all of the study participants had heard of *Shigella*, most did not view a standalone *Shigella* vaccine as being of high priority. Perceived priority increased when participants were provided information about *Shigella*’s link with growth stunting and AMR, but there remained considerable uncertainty about the true burden of *Shigella* and the impact of a standalone vaccine. A combination vaccine (and/or oral formulation) was clearly considered desirable [15].

**Fig. 1:**
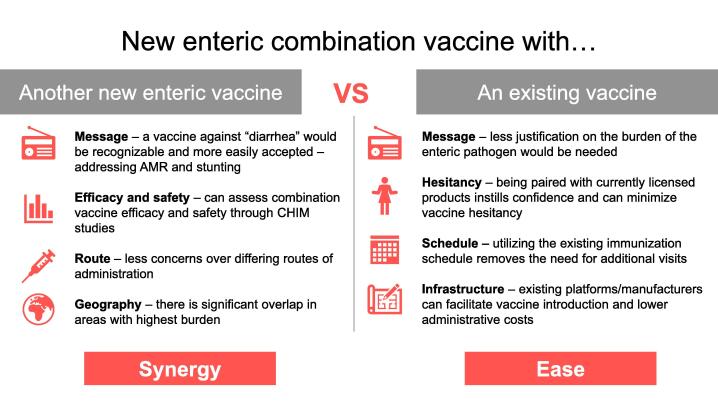
Key points from small group discussions on potential new combination vaccines (an all-enteric pathogen combination vs. combining a new enteric vaccine with an existing EPI vaccine), as summarized by Madison Billingsley and Bettina Wunderlich, both of Johns Hopkins University Bloomberg School of Public Health.

Primed by these presentations, the workshop participants broke into two groups. One group discussed the merits of a combination formulation comprising all enteric pathogens that are not currently part of the existing Expanded Programme on Immunization (EPI) schedule, which might have a greater synergistic impact on the syndromes of diarrhea and dysentery, as well as AMR. The other group discussed the advantages of a combination approach where the enteric vaccine is combined with an existing EPI vaccine, which might provide the easiest way forward. The highlights of these deliberations are depicted in Fig. 1.

## 3 New tools for the development and evaluation of vaccines

### 3.1 Integrating antigen/antibody technologies for mucosal vaccine and immunoprophylactic development

#### Organizers: Nicholas Mantis (Wadsworth Center, New York State Department of Health); Joe Campo (Antigen Discovery, Inc.)

This workshop highlighted emerging antigen and antibody technologies which together form an integrated pipeline to accelerate discovery of novel antigens and the subsequent development of enteric vaccines and immunoprophylactics. The first speaker, Joe Campo, described the use of high-density antigen arrays consisting of proteins, polysaccharides, and even cell lysates to profile multi-isotype antibodies from serum and other sample types, such as breast milk, lymphocyte supernatants, and fecal extracts [16], [17]. The high-density arrays were able to identify antibody reactivity profiles associated with age, natural infection, vaccination, adjuvants and, in some cases, the elicitation of on-target and off-target responses [18]. One important takeaway was that antigen-specific IgA and IgG responses vary within and among populations so much that a single type of response (e.g., anti-protein or anti-LPS) may be insufficient to distinguish between protected and susceptible disease status, underscoring the need for broader immune profiling and account for different cohort make-up when defining correlates of protection and anticipated impact of vaccines on disease incidence.

As a complementary approach to antigen discovery, Omar Rossi (GSK Vaccines Institute for Global Health) described advances in reverse vaccinology. In its original iteration, Reverse Vaccinology (RV) involved computational mining of a pathogen’s genome for putative vaccine antigens based on the propensity of a given open reading frame to encode for surface-exposed, secreted, and/or virulence factors. Dr. Rossi described a new era of reverse vaccinology (RV2.0) driven by high-throughput screening of human monoclonal antibodies (huMAbs) from vaccinated or convalescent individuals or controlled human infection model (CHIM) subjects for reactivity with a desired target antigen or with *in vitro* functional activity (e.g., bactericidal). When coupled with high-resolution epitope mapping methodologies and *in vitro* functional assays, he explained how the characterization of large panels of huMAbs can uncover conformational and broadly cross-reactive epitopes that may serve as basis of vaccine antigens. Dr. Rossi described how the approach has been applied to the discovery of therapeutic MAbs against different strains of *Shigella*, which involved screening more than 40,000 memory B cells for huMAbs with opsonophagocytosis killing activities, reactivity with a 90-plex Luminex panel, and inhibitory adhesion and invasion activities.

The issue of how to interrogate large immunological data sets and derive correlates of protection associated with mucosal vaccines was addressed by Galit Alter (Ragon Insititute; Harvard Medical School). Dr. Alter described the application of systems serology to identify serum-based correlates of immunity to *Shigella* in a cohort of healthy individuals that had been orally vaccinated with a candidate *Shigella* vaccine [19]. The study revealed the power of machine learning and multivariate analysis in identifying a significant correlation between IpaB-specific serum antibody levels and reduction in disease severity in the CHIM. The capacity to quantitate functional activities such as antibody-dependent neutrophil phagocytosis was central to parsing out key correlates of mucosal immune mechanisms. A central theme presented by Dr. Alter was the stark contrast in immune responses between non-endemic and endemic populations. Protective responses that are common across populations, such as anti-*Shigella* IgA driven FcαR binding and functions, may be better predictors of protection than antibody levels alone.

Francesca Micoli (GSK Vaccines Institute for Global Health) described the evolution of Generalized Modules for Membrane Antigens (GMMA) from an antigen discovery strategy to a platform for bacterial vaccines, including systemic (meningitis) and enteric infections, namely *Salmonella enterica* serovars Typhimurium and Enterica. The benefits of GMMA over conventional live attenuated, heat killed, or conjugate vaccine platforms were enumerated, and included affordability, self-adjuvanting properties, and capacity to readily engineer bacterial strains to elicit GMMA with antigenic cargo of interest. Dr. Micoli also described the use of GMMA to identify novel cross-reactive antigens to identify vaccine candidates between related pathogens such as meningitis and gonorrhea.

Finally, Nicholas Mantis showcased a recent study demonstrating the use of human intestinal enteroids and organoids to recapitulate IgA transcytosis and enteric immunity to *S*. Typhimurium. The investigators demonstrated basolateral to apical transport of dimeric Sal4 human IgA2 (but not monomeric Sal4 IgA2) that was dependent on expression of the polymeric immunoglobulin receptor (pIgR). Sal4 IgA transport across both enteroid and organoid-derived monolayers reduced invasion by *S.* Typhimurium in a dose-dependent manner. Sal4 IgG also conferred a degree of protection that was proposed to be mediated by the neonatal Fc receptor (FcRn). This study opens the door to the use of intestinal enteroid and organoids as tools to evaluate enteric vaccines and immunoprophylactics against a range of invasive and non-invasive pathogens.

### 3.2 The need for adjuvants for *Shigella* vaccines to ensure protective efficacy among children in LMICs

#### Organizer: Calman A. MacLennan (Bill & Melinda Gates Foundation)

Calman MacLennan introduced the workshop, explaining the need to consider adjuvants to mitigate a possible lack of protection in the target population of young children in LMICs with *Shigella* vaccines. He summarized outcomes from a *Shigella* adjuvants workshop in 2020 which concluded that possible adjuvants should be prioritized based on ability to formulate, enhancement of conjugate vaccine immunogenicity, and cost. The need to test *Shigella* vaccines formulated with adjuvants other than aluminum salts in animal models was articulated. Though use of adjuvants included in licensed vaccines was generally preferred, squalene-in-water emulsion (SWE) and double-mutant heat-labile enterotoxin (dmLT), which are not in licensed vaccines, had most support from attendees.

Laura Martin (US Pharmacopeial Convention) reviewed recent clinical trial data comparing *Shigella* vaccines formulated with and without aluminum hydroxide. Phase 1 studies with monovalent *Shigella flexneri* 2a conjugate vaccines, Flexyn 2a (LimmaTech Biologics AG), and Sf2a-TT (Institut Pasteur), while not powered to demonstrate an adjuvant effect and usually with no impact of aluminum salt observed, gave a signal of increased serum O-antigen IgG with aluminum adjuvant at low vaccine dose. Addition of aluminum salt also shifted the IgG class elicited. Intriguingly, a Phase 2 study with the bivalent ZF091 *Shigella* conjugate vaccine (Beijing Zhifei Biopharmaceutical Company) demonstrated increased serum IgG to *S. flexneri* 2a O-antigen but decreased IgG to *S. sonnei* O-antigen when formulated with aluminum hydroxide.

Aurelio Bonavia (Bill & Melinda Gates Medical Research Institute) reviewed the changing landscape of novel adjuvants which has advanced rapidly with the Covid-19 pandemic. CpG 1018 + aluminum salt, Matrix M, and Alhydroxiquim-II were discussed. However, translating findings from these adjuvants with SARS-CoV-2 vaccines to *Shigella* conjugate vaccines is not straightforward, and there is considerable uncertainty whether they will benefit *Shigella* vaccines. Encouraging results have been observed in non-human primates with pneumococcal and *Campylobacter* conjugate vaccines that included 3M-052AF/aluminum salt and Army Liposome formulation containing QS 21 saponin (ALFQ), respectively.

In subsequent discussion, the consensus preference for new *Shigella* vaccines was an adjuvant that has a large safety database and ideally one included in a pediatric licensed vaccine formulation. It was uncertain whether an adjuvant other than an aluminum salt would be safe and efficacious in the target population. Though this will partly be answered by current studies of *Shigella* vaccine formulations in African children, the group appreciated the lost opportunity with delaying testing of alternative adjuvants until new clinical data are available.

Participants considered it valuable to evaluate new *Shigella* candidate vaccine formulations with novel adjuvants head-to-head with aluminum salt and unadjuvanted formulations in preclinical animal models, while appreciating some adjuvants might not have commercial-scale processes. Resulting data will be crucial for regulatory approval for testing of new adjuvants with *Shigella* vaccines in humans. Serum O-antigen IgG is important given its correlate of protection status, but other immune parameters should also be examined.

Despite a preference for adjuvants in licensed vaccines, evidence of positive impact with 3M-052-AF + aluminum salt and ALFQ highlighted these for preclinical testing. Advancing the most promising adjuvant-vaccine combinations to human trials is particularly important. The key message was that planning of adjuvant studies, if not already started, needs to begin now.

## 4 Emerging enteric pathogens

### 4.1 Invasive non-typhoidal salmonellosis: Developing a vaccine against a neglected disease

#### Organizer: Maheshi Ramasamy (University of Oxford)

Non-typhoidal *Salmonellae* most commonly cause a self-limiting gastroenteritis that is clinically indistinguishable from that caused by many other enteric pathogens. However, these bacteria can also cause an invasive syndrome (iNTS) with bacteremia, fever, and metastatic infection, which if untreated can lead to septicemia and death [20]. *Salmonella* Enteritidis and *Salmonella* Typhimurium (STm) represent the most common serovars to cause invasive disease [20]. STm isolates with the multi-locus sequence type ST313 have emerged as a dominant subtype in sub-Saharan Africa. Genomic analysis of these invasive isolates reveals genome degradation and pseudogene formation, characteristics consistent with host adaptation and restriction [21]. Along with enhanced invasive and virulence factors, the ST313 subtype is associated with multidrug resistance, potentially contributing to higher incidence and mortality from iNTS in sub-Saharan Africa. Development of an effective and affordable vaccine against iNTS is thus an essential control measure against this neglected disease. Animal vaccines based on attenuated organisms are in widespread use in domestic livestock and have been successful at reducing food-borne gastroenteritis.

Several human vaccine candidates have been developed against iNTS, but all remain in preclinical or Phase 1 studies:

• A trivalent glycoconjugate vaccine developed by the University of Maryland consisting of STm and SEn O antigen conjugated to homologous FliC flagellar protein combined with *S.* typhi Vi polysaccharide conjugated to tetanus toxoid has demonstrated robust antibody responses in a Phase 1 trial. A further trial is planned with a larger sample size and different dose regimes.
• A vaccine based on outer membrane vesicles (GMMAs) developed by GSK Vaccines for Global Health is currently in Phase 1 trials in the United Kingdom with a further study planned to start in an endemic Kenyan population later in 2023. Trials are also planned with a trivalent formulation incorporating a *S.* typhi Vi-CRM conjugate.
• A multiple antigen presentation system (MAPS) based vaccine developed by Affinivax uses biotin/rhizavidin interactions to link STm and SEn O antigens to SseB type 3 secretion system protein and demonstrates humoral and cellular immune responses in animal models. A quadrivalent vaccine additionally incorporating *S.* typhi Vi polysaccharide and *S.* paratyphi O antigen linked to Cp1 pneumococcal protein is also in preclinical studies.

An effective bivalent STm/SEn vaccine would clearly target iNTS disease in children in LMICs. However, incorporating a typhoid conjugate vaccine to create a trivalent vaccine (or a paratyphoid vaccine in the future to create a quadrivalent vaccine) may facilitate integration into existing EPI schedules.

The acquisition of serum bactericidal activity with age appears to be associated with lower incidence of invasive disease, but the relative importance of humoral and cellular responses in protection remains uncertain [22]. In the absence of a known correlate of protection, large-scale field trials would be needed to test efficacy of any new iNTS vaccine. A novel CHIM of iNTS is planned at Imperial College London in 2023, using both diarrhoeagenic and invasive strains of STm. This will provide insights into immune responses to infection and potentially provide an alternative platform to test the efficacy of future iNTS vaccine candidates.

### 4.2 Prevention strategies to defeat *Campylobacter*

#### Organizer: *Frédéric* Poly (US Naval Medical Research Command [NMRC]

This workshop focused on the prevention of *Campylobacter* among children in LMICs. James Platts-Mills (University of Virginia) first summarized the latest studies on the impact of *Campylobacter* infection in this population. Parenteral vaccine strategies targeting *Campylobacter* were presented by Renee Laird (NMRC) and the development of a blue-green algae passive oral immunoprophylaxis was presented by Ben Jester (Lumen Bioscience). The workshop provided an opportunity for participants to debate the most appropriate prevention measures against *Campylobacter*.

*Campylobacter* has been recognized since the mid-1970 s as a causative agent of diarrhea in humans. *Campylobacter* burden has been underestimated due primarily to the difficulties of isolation from human stools [23]. The recent development and implementation of molecular and immunological assays for the detection of *Campylobacter* in stools remediate the isolation constraints [24]. Surveillance in high-income countries demonstrates that *Campylobacter* is the primary cause of bacterial foodborne disease in the United States and Europe. In LMICs, *Campylobacter* incidence is one of the most common diarrhea etiologic agent in infants (0–11 months of age) in both the Global Enterics Multicenter Study (GEMS) and Malnutrition and Enteric Disease Study (MAL-ED) [24], [25]. In children 1–4 years of age, *Campylobacter* remains frequently identified as sub-clinical cases of infections. In these settings, *Campylobacter* asymptomatic infections appear to have a dramatic impact on stunting [26].

In the last three decades, several vaccines against *C. jejuni* have been clinically evaluated [27]. The latest iteration, a polysaccharide conjugate prototype vaccine developed by NMRC is currently being evaluated in a Phase 1 trial. The vaccine was developed with an adult population in mind (military, travelers). Due to the impact of *Campylobacter* on diarrheal disease in infants, a *Campylobacter* vaccine would have to be provided to an early age. Unfortunately, the infant vaccination schedule is already crowded. The inclusion/acceptance of an additional vaccine by government health agencies is unlikely. The development of a pan-diarrhea vaccine that includes the most common sources of pediatric diarrheal disease including *Shigella*, ETEC, rotavirus, and *Campylobacter* would likely increase acceptance. Oral passive immunoprophylaxis appears to be a viable alternative to vaccines for infants as well as toddlers in LMICs. The implementation of a low-cost food supplement containing immunoglobulins targeting *Campylobacter* such as the spirulina, a blue-green algae bioengineered to produce antibodies targeting *C. jejuni* flagella have the potential to limit pathogen colonization, acute disease, and associated sequalae [28].

The workshop discussion recognized the importance of *Campylobacter* as a source of pediatric infections leading to diarrheal disease, morbidity, stunting, and subsequent impact on social and economic development in LMICs. Efforts towards advocacy and education on the burden of *Campylobacter* to LMICs governmental health agencies needs to be increased in order to enhance the chance for introduction of either passive and or active prophylactic measures to defeat *Campylobacter.*

## 5 The assessment of vaccine impact on acute and long-term morbidity

### 5.1 Characterizing the nature and severity of intestinal inflammation to guide enteric vaccine and prophylactic product development and evaluation

#### Organizers: Lou Bourgeois (PATH); Shahida Baqar (US National Institutes of Health)

This workshop had two goals. The first was to assess progress in the development of assays that may lead to better tools for measuring inflammatory biomarkers in infants and children living in LMICs. These are important because infection with enteric pathogens can lead to significant inflammation in early childhood that is a probable risk factor for enteric environmental dysfunction (EED), stunting, and poor cognitive development [29], [30], [31], [32]. The second goal was to better understand the impact of inflammation associated with vaccination or enteric infections. Failure to strike the appropriate balance between vaccine-induced inflammation and safety, tolerability, and immunogenicity can interfere with successful immunization.

Field studies have shown that even asymptomatic ETEC infection can cause significant intestinal inflammation [29]. However, these studies are often confounded by coinfections making it difficult to determine the sole impact of the ETEC infection. In contrast, CHIMs enable more causal relationships to be evaluated with subjects challenged with a single ETEC strain [33]. As presented by Subhra Chakraborty (Johns Hopkins Bloomberg School of Public Health), symptomatic and asymptomatic infection with ETEC strain H10407 led to significant challenge dose-dependent elevations in fecal myeloperoxidase (MPO) and serum intestinal fatty acid binding protein (I-FABP), findings consistent with earlier field data [33]. These CHIMs-based observations strengthen the case that ETEC strains can contribute to the inflammatory pathway leading to stunting and EED [29], [33].

A serum-based multiplex assay, the micronutrient and environmental enteric dysfunction assessment tool (MEEDAT), has been used to measure 11 biomarkers associated with stunting and EED [34]. Fecal extracts from participants in a *Campylobacter jejuni* CHIMs were assessed for levels of inflammatory markers using immunosorbent assays for MPO, Lipocalin-2, and several cytokines. As presented by Heather Eggleston (NMRC), serum concentrations of C-reactive protein (CRP), alpha-1-acid glycoprotein (AGP), insulin-like growth factor 1 (IGF-1), and I-FABP were assessed using MEEDAT. Subjects received an initial challenge with strain 81–176 and were re-challenged approximately 1 month or 1 year later with the homologous strain [35], [36]. *C. jejuni* caused significant inflammation in the CHIMs, yet inflammation was significantly blunted in re-challenged volunteers with protection waning over time. Concentrations of fecal MPO were strongly correlated with serum CRP and AGP, suggesting that gut and systemic inflammation were linked and that MEEDAT can provide useful insight.

Innovative results from the Walter Reed Army Institute of Research indicated that the msbB mutation results in an underacylation of the Lipid A portion of the *Shigella* LPS and reduces its endotoxicity. As presented by Malabi Venkatesan (independent consultant), such a detoxified LPS improves the safety and tolerability of both live attenuated, oral and parenteral LPS-based subunit *Shigella* vaccines without impacting immunogenicity [36], [37]. For example, intramuscular immunization of guinea pigs with the Invaplex_AR-DETOX_ candidate subsequently challenged by the rectocolitis model with *S. flexneri* 2457 T prevented acute diarrhea and dysentery and blunted the intestinal MPO response. Invaplex_AR-DETOX_ exemplifies WHO’s new strategic goals for *Shigella* vaccines in that it significantly impacts on both acute disease and occurrence of inflammation possibly associated with long-term sequelae.

Ed Ryan (Harvard T.H. Chan School of Public Health) presented comparative field data that were used to evaluate the relationship between inflammation, EED, and effective immunization of Bangladeshi infants and young children with an oral, inactivated whole cell cholera vaccine (OCV), live attenuated oral vaccines (LAVs) against rotavirus and polio, and several parenteral EPI vaccines [38], [39]. In these studies, inflammatory biomarkers of EED were associated with vaccine failure or underperformance of the LAVs, but were positively associated with better immune responses to OCV, suggesting EED’s impact on LAVs and OCVs may differ. Elevations of intestinal biomarkers had no impact on the immunogenicity of parenteral EPI vaccines.

There was general agreement among the workshop participants that, given the high global burden of EED and stunting, continued evaluation of MEEDAT and other new tools for characterizing inflammation are needed to improve understanding of the complex interplay between intestinal and systemic inflammation that leads to poor health outcomes in young children. With this in mind, Michael Arndt (University of Washington) recommended that future enteric vaccine trials consider risk-stratifying children with biomarkers of inflammation. The general consensus was that it is too early to focus on only one or two inflammatory biomarkers, as many new potential contributors, such as IL-33, have only recently been identified [40]. Ultimately, an index of multiple biomarkers may be needed to adequately characterize the risk of negative health outcomes and suboptimal immunization responses associated with aberrant gut and systemic inflammation. In addition, a vaccine that reduces both the risk of acute illness and inflammation may have a greater public health impact.

### 5.2 Cellular immunity and immunological memory in ETEC and *Shigella* infections and vaccination in humans

#### Organizers: Kurt Hanevik (University of Bergen); Marcelo B. Sztein (University of Maryland)

Our understanding of the kinetics and functioning of immune cell subsets in protection from ETEC and *Shigella* remains inadequate. The role of T-cell and B-cell subsets needs to be better defined and their association with protection assessed. This knowledge can be crucial in guiding development of next-generation vaccines able to modulate the immune response toward durable protection. This workshop explored this understudied area in ETEC and *Shigella* infections.

Anna Lundgren (University of Gothenburg) presented data that ETEC vaccination induces both antibody in lymphocyte supernatant (ALS) and memory B cell responses to vaccine colonization factors and LTB, and that frequencies of plasmablasts correlate with vaccine specific IgA ALS responses [41]. It has been found that B Cell Maturation Factor (BCMA) measured in ALS reflected the total plasmablast response, that ALS BCMA levels indicated dmLT adjuvant effects, and that ALS BCMA levels after primary vaccination predicted the vaccine specific memory B cell (Bm) response [42]. Consistent data support the importance of circulating T follicular helper (cTfh) cell responses in both ETEC vaccination and natural infection.

Kurt Hanevik presented high-dimensional mass cytometry data of lymphocyte kinetics during an experimental ST-only ETEC infection model, identifying 27 innate and adaptive lymphocyte populations [43]. Subsets of mucosal-associated invariant T (MAIT) cells decreased, while natural killer cells and dendritic cells tended to rise at day 3. An increase in plasmablasts on days 5–7 was paralleled by a gradual increase in Th17-like effector and central memory and regulatory subsets. The Th17-like subsets showed increased proliferation and expression of activation and gut-homing markers. These responsive Th17-like cell subsets increased at an earlier timepoint in the non-diarrhea group suggesting a recall response (S. Rim et al., in press)*.*

Building on previous data, Daniel Cohen (Tel Aviv University) discussed new short- and long-term B memory cells (Bm) and antibody responses elicited in humans following immunization with a synthetic carbohydrate-based conjugate vaccine against *Shigella flexneri* 2a (SF2a-TT15) [44]. Immunization resulted in elevated serum IgG, IgA, and IgM antibodies to SF2a LPS, serum bactericidal antibodies (SBA), improved avidity, and specific IgG and IgA ASC and Bm responses. Novel data on long-term immunity showed that serum IgG and IgA to LPS, SBA, and avidity, remained elevated up to three years after vaccination. Importantly, although circulating IgG and IgA Bm cells declined over time, levels of circulating IgG Bm cells early after immunization predicted antibody levels two years later.

Franklin Toapanta (University of Maryland) presented novel data on the identification and characterization of *Shigella*-specific Bm cells after vaccination with SFa2a-TT15 [44] using a newly developed flow cytometry-based technique involving dual staining with SF2a bacteria labeled with either of two different fluorochromes [44]. Specific switched Bm cells increased after vaccination and expressed high levels of CXCR3 and low levels of CD21. Correlations were observed between the frequencies of these cells and anti-LPS IgG and IgA antibodies in serum, supporting and extending findings in the Phase 1 study [44]. Finally, novel data were presented using polyclonally expanded B cells which replicated the data obtained with ex-vivo Bm cells.

### 5.3 Clinical and microbiologic endpoints for *Shigella* vaccine efficacy studies in children in LMICs

#### Organizers: Patricia Pavlinac (University of Washington); Richard Omore (Kenya Medical Research Institute); Elizabeth T. Rogawski McQuade (Emory University); Chad Porter (NMRC)

Several candidate *Shigella* vaccines are advancing toward necessary efficacy evaluations in children in LMICs. In this workshop, presenters reviewed data and recent consensus statements regarding endpoints for pivotal efficacy studies of *Shigella* vaccines for this population. Various clinical and microbiological endpoints were examined with consideration of the sample size implications. The framework for the discussion was that a primary goal of a pivotal efficacy trial should be to estimate the efficacy of a *Shigella* vaccine administered to young children against diarrheal episodes caused by antigenic types contained in the vaccine.

Breakout groups discussed advantages and disadvantages of various definitions for the primary clinical endpoint, with a general agreement on the need to focus on moderate or severe shigellosis. The main options considered were: 1) moderate-to-severe diarrhea (MSD) defined as dysentery, dehydration, or hospitalization as in GEMS, and 2) severe or moderate + severe diarrhea defined by cut-points from a Vesikari score modified to incorporate dysentery. Advantages of the GEMS MSD definition include its use across recent diarrhea etiology studies, ability to determine severity at the point of presentation, and demonstrated association with poor outcomes. The modified Vesikari definition adds increased flexibility to estimate vaccine efficacy for subsets of severity defined by various score cut-offs and captures severity of the full diarrhea episode. It is also widely used and accepted in pediatric rotavirus trials with the inclusion of dysentery capturing intestinal manifestations of colitis often associated with shigellosis.

Breakout group discussions regarding diagnostic approaches focused on traditional culture compared to molecular diagnostics. Isolating *Shigella* in diarrheal episodes by culture enables serotyping and antibiotic susceptibility testing with clear clinical relevance. However, molecular diagnostic methods are more sensitive, enabling smaller sample size requirements and have recently become able to perform serotyping.

Workshop participants agreed that the effects of a *Shigella* vaccine on linear growth faltering, mortality, and antimicrobial use and resistance also need to be understood. If sample sizes permit, these outcomes could be included as secondary or exploratory endpoints of a Phase 3 trial, or otherwise could be evaluated during post-licensure effectiveness studies.

### 5.4 Intricacies of protective immunity to enteric pathogens

#### Organizers: Armelle Phalipon (Institut Pasteur); Marcela Pasetti (University of Maryland); Robert Kaminski (Latham BioPharm Group)

For many enteric pathogens, immunity derived from a repeated prior exposure renders an individual resistant to subsequent infection and/or disease. For this reason, vaccine development efforts largely have been focused on understanding and then replicating comparable immune responses through vaccination. However, rational design of vaccines to induce protective immunity requires a more complete understanding of the intricacies of this naturally acquired immunity as well as immunity elicited by immunization approaches that have been successful in preventing disease caused by other mucosal pathogens. The workshop’s goal was to review current knowledge and insights on immunity against enteric pathogens, vaccine development, host-pathogen interactions, and early-life immunology. Experts in the field outlined the intricacies of protective immunity against enteric pathogens, and specific gaps in knowledge for focused discussions and future research that can inform vaccine development.

Discussions were centered around presentations by James Fleckenstein (Washington University, St. Louis), Marcela Pasetti, and Miren Iturriza-Gomara (PATH). Dr. Fleckenstein provided an overview of ETEC molecular pathogenesis, preclinical vaccinology focused on ETEC vaccine development, and the identification of novel antigens as potential vaccine candidates, such as EtpA adhesin and EatA mucinase. He also highlighted ETEC toxins that can act as potential drivers of enteropathy and suggested these targets such as LT for vaccine-induced immunity.

Dr. Pasetti focused on the immune responses in infants, who represent the target population for most enteric vaccines outside traveler/military populations. She highlighted the contribution of breast milk and the need to discern the coordinated role of immune effectors, including serum IgA, mucosal antibodies, and their FcRc engagement of innate immune cells. Subsequent discussion included invasiveness/inflammation and induction of immunity by infection and vaccination, potential markers, and predictors of clinical protection (LPS IgG, ALS Ab), thresholds of protection, and standardization of assays, as well as availability and common use of reagents and standards.

Dr. Iturriza-Gomara provided an intriguing overview of immune responses elicited by oral rotavirus vaccines and associated with reduced shedding and illness. She explained that rotavirus-specific IgA antibodies are associated with protection, even in the absence of neutralizing activity. The role of rotavirus IgG is understudied, but data from passive immune therapy and maternally derived protection suggest it is likely to be important, and B cells carrying the α4β7 intestinal homing receptor were highlighted as important for rotavirus clearance and protection from reinfection. Breast milk components related to vaccine take/shedding was again a topic of discussion, in the context of rotavirus and other enteric vaccines.

Overall, the workshop highlighted the continued need to advance the availability and use of reagents and standards to allow the global community to better interrogate protective immune mechanisms, the need for additional immunogenic targets for vaccine development, and the complexities and elusiveness of immunity to enteric pathogens.

## 6 Conclusion

This summary of the 2022 VASE Conference workshop proceedings reflects the breadth and depth of topics explored. These sessions, which depend on robust audience participation and discussion, brought together diverse inputs from experts working in different disciplines around the world. As evidenced by the summaries above, these small-group sessions allowed the attendees to learn about the latest research, as well as new tools aiding enteric vaccine development, while also holding in-depth discussions about specific topics important to advancing critical new concepts to the enteric vaccine field. The collective outputs from these workshops point to the value they add to the enteric vaccine field and the benefits of workshop participants seeing their contributions to critical topics in real-time.

## 7 Funding

This work was supported by the Bill & Melinda Gates Foundation [INV-000875]. Under the grant conditions of the Foundation, a Creative Commons Attribution 4.0 Generic License has already been assigned to the Author Accepted Manuscript version that might arise from this submission.

## 8 Copyright statement

Some of the authors are U.S. Government employees. This work was prepared as part of their official duties. Title 17 U.S.C. §105 provides that ‘‘Copyright protection under this title is not available for any work of the United States Government.” Title 17 U.S.C. §101 defines a U.S. Government work as a work prepared by a military service member or employee of the U.S. Government as part of that person’s official duties.

## 9 Disclaimer

The opinions expressed in this article are those of the authors and do not reflect the view of the Department of the Navy, Department of Defense or the United States Government. There are no restrictions on its use.

## Declaration of competing interest

The authors declare that they have no known competing financial interests or personal relationships that could have appeared to influence the work reported in this paper.

## Data Availability

This is a conference report and does not contain original data.
